# Bioactive components and delivery strategies of food-medicine homologous substances in lung cancer therapy: current advances and clinical translation

**DOI:** 10.3389/fnut.2025.1696289

**Published:** 2025-12-15

**Authors:** Man Sun, Dan Zang, Jun Chen

**Affiliations:** Department of Oncology, The Second Hospital of Dalian Medical University, Dalian, Liaoning, China

**Keywords:** food-medicine homologous, nutritional intervention, lung cancer, gut–lung axis, nanocarriers, review

## Abstract

Lung cancer remains the leading cause of cancer-related mortality worldwide, challenged by drug resistance, treatment toxicities, and limited efficacy. Recently, food-medicine homologous (FMH) substances have gained attention as adjunctive therapies due to their multi-target effects, low toxicity, and favorable safety profiles. As the “food as medicine” paradigm integrates into health policies, a comprehensive understanding of FMH in lung cancer therapy as a form of nutrition-based adjuvant therapy is increasingly relevant and timely. This review summarizes the anti-lung cancer mechanisms of FMH-derived bioactive compounds—such as polysaccharides, saponins, polyphenols, alkaloids, and essential oils—focusing on their roles in cell cycle regulation, apoptosis induction, immune modulation, and resistance reversal. To address poor bioavailability, we highlight recent advances in nanodelivery systems that enhance therapeutic efficacy. Moreover, we examine nutritional modulation of gut microbiota under the gut–lung axis framework as a novel strategy in lung cancer treatment. By integrating preclinical evidence with translational studies, this review evaluates the clinical potential and future directions of FMH-based therapies. The aim is to provide a theoretical basis for optimizing integrative Chinese–Western approaches and advancing personalized nutrition-oriented strategies in lung cancer care.

## Graphical abstract

**Figure:**
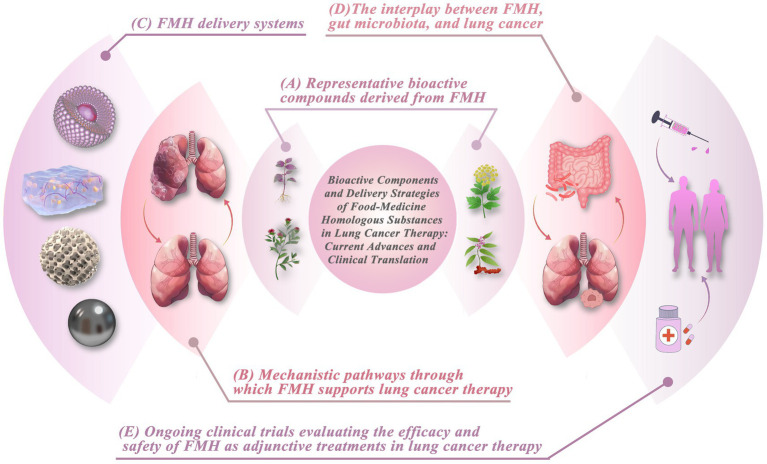

## 1 Introduction

Lung cancer continues to represent a significant challenge to global public health, with both prevention and treatment facing substantial obstacles. According to the most recent global cancer statistics published in 2024, lung cancer is responsible for over 2.2 million new cases and 1.8 million deaths each year, solidifying its status as the leading cause of both incidence and mortality among all malignancies (1). This concerning trend is closely linked to risk factors such as aging, prolonged tobacco exposure, environmental pollution, and chronic lung damage from COVID-19 (2, 3). Despite breakthroughs in targeted and immune therapies, three major challenges persist in clinical practice: acquired drug resistance, inadequate predictive biomarkers, and significant treatment-related toxicities (4). Therefore, developing strategies that integrate high efficacy, low toxicity, and resistance-overcoming capabilities is imperative to improve clinical outcomes.

Amid progress in precision medicine, nutrition-oriented, low-toxicity interventions based on the concept of food-medicine homologous (FMH) substances have attracted increasing attention. Recognized by the World Health Organization as a component of nutritional strategies for cancer prevention, FMH have been gradually integrated into oncological care (5). These substances offer dual nutritional and pharmacological benefits by regulating immune and metabolic homeostasis, and their long-standing use in dietary therapy underscores their safety and nutritional relevance (6). According to the primary classical source Huangdi Neijing (The Yellow Emperor’s Inner Canon) records, “When consumed by the healthy, they serve as food; when consumed by the ill, they serve as medicine,” highlighting the dialectical unity between food and medicine (7, 8). This concept aligns with the modern understanding of functional foods and nutraceuticals as mediators of disease modulation through dietary means.

FMH have demonstrated antitumor potential in various malignancies, including breast and colorectal cancers, with several agents entering clinical evaluation, indicating promising applications in lung cancer. Currently, 106 FMH substances—such as Astragalus, Ginseng, and *Lycium chinense*—have been identified for their bioactive and nutrition-linked properties. Bioactive compounds, including curcumin, ginsenosides, and *Ganoderma lucidum* (Curtis) P. Karst. (Lingzhi mushroom) polysaccharides, exert anti-cancer effects through apoptosis induction, anti-angiogenesis, immune modulation, and regulation of gene expression. These mechanisms contribute not only to enhanced treatment efficacy and resistance reversal but also to nutritional support and metabolic balance restoration (9). Importantly, FMH-based interventions can improve nutritional status, reduce treatment-related side effects, and promote quality of life, thereby supporting their integration into nutrition-guided and personalized lung cancer therapy.

Although interest in FMH is increasing, systematic reviews on their mechanisms, delivery strategies, and clinical translation in lung cancer remain limited. This narrative review provides an integrative summary of recent advances in FMH research, focusing on nutritionally relevant bioactive compounds, extraction and synthesis techniques, pharmacological mechanisms, and innovations in nanodelivery systems that enhance bioavailability. Additionally, it explores the modulation of the gut–lung axis as a novel nutrition-linked therapeutic strategy. By integrating experimental and clinical findings, this review aims to offer a theoretical foundation and practical guidance for translating FMH-based nutrition-based adjuvant therapy into lung cancer prevention and treatment frameworks. Given the evolving and interdisciplinary nature of this field, this review is based on a targeted synthesis of recent literature rather than a predefined systematic review protocol. Relevant studies (2000–2024) were identified through PubMed, Web of Science, and Scopus using standardized FMH, lung cancer, and gut–lung axis keywords.

## 2 FMH-derived bioactive agents for lung cancer adjuvant treatment

Bioactive compounds from FMH—such as polysaccharides, saponins, polyphenols, alkaloids, and essential oils—show promising potential as nutritional adjuvants in lung cancer therapy. These five categories were selected based on their high frequency in recent FMH literature, well-characterized pharmacological mechanisms, and translational relevance across both food and medicinal contexts. Beyond their pharmacological effects, these compounds support nutritional modulation of immune and metabolic pathways. This section summarizes their major anticancer mechanisms, immunometabolic modulation, and translational relevance in lung cancer.

Notably, the applicability of FMH-derived compounds may vary between lung cancer subtypes. NSCLC and SCLC differ significantly in terms of molecular profiles, immune microenvironments, and therapeutic responsiveness. Existing studies have primarily focused on FMH effects in NSCLC models, where immune modulation and metabolic reprogramming are more pronounced. In contrast, evidence on FMH efficacy in SCLC remains limited, partly due to its aggressive neuroendocrine phenotype and poor immunogenicity. Future research should therefore consider tailoring FMH-based interventions according to histological subtype and molecular vulnerabilities.

### 2.1 Polysaccharide-based therapeutic agents

Polysaccharides, as macromolecular compounds composed of monosaccharide units, are widely present in FMH such as Polygonatum, *Ganoderma lucidum* (Curtis) P. Karst. (Lingzhi mushroom), and Lentinula edodes (10). For example, β-glucan-rich polysaccharides from *Lentinula edodes* enhance T cell, macrophage, and NK cell activity, boosting cellular immunity. Injectable forms like “Tiandixin” have been approved for adjuvant therapy in gastric and lung cancers (11). *Astragalus* polysaccharides (PG2) inhibit tumor growth by activating M1 macrophages, enhancing Th1 responses, reducing Tregs, and modulating Wnt/β-catenin and RAS/ERK pathways; PG2 is under development for antitumor use (12). *Panax* polysaccharides, rich in arabinose and galactose, have strong antioxidant effects and are formulated into tablets and capsules to reduce chemotherapy-induced oxidative stress (13). These polysaccharides are evolving from traditional remedies to standardized pharmaceuticals, with several in clinical use, marking a major step in their application as effective cancer adjuvants.

Clinically, polysaccharides are used as adjuvants in chemo- and radiotherapy to enhance immunity and reduce side effects, as seen with *Coriolus*, *Ganoderma*, and *Lentinula*-based formulations. Further mechanistic studies will support their therapeutic development (14). In-depth investigation of their pharmacological mechanisms will facilitate the advancement of polysaccharide-based therapeutics in the field of cancer treatment.

### 2.2 Saponin-based therapeutic agents

Saponins, consisting of hydrophilic sugar chains and hydrophobic sapogenins, are abundant in FMH such as *Panax ginseng*, Astragalus membranaceus, and Glycyrrhiza uralensis (15). Based on sapogenin structures, they are categorized into steroidal (e.g., diosgenin, ophiopogonins) and triterpenoid types (e.g., ginsenosides, astragalosides), exhibiting broad pharmacological activities including anticancer potential. Ginsenosides, particularly Rg3 in Shenyi Capsule—recommended by NCCN Guidelines (2016) for NSCLC—suppress proliferation and support chemotherapy (16). Astragalosides and diosgenins modulate immunity and hormone metabolism, with formulations like Jinshuibao Capsule demonstrating immune and anticancer activity (17). Mechanistically, saponins inhibit tumor proliferation, induce apoptosis, and suppress angiogenesis and metastasis (18).

### 2.3 Polyphenol-based therapeutic agents

Polyphenols are natural compounds with aromatic rings and hydroxyl groups, found in FMH plants like *Pueraria lobata*, *Scutellaria baicalensis*, and *Ginkgo biloba* (19). Structurally, they include flavonoids, phenolic acids, lignans, and stilbenes—of which flavonoids (~60%) and phenolic acids (~30%) are the main anticancer agents (20). Their biological effects depend on botanical origin, for instance, isoflavones from *Pueraria lobata* exhibit phytoestrogenic activity (21). Baicalin, known for anti-inflammatory and pro-apoptotic effects, is included in antitumor formulations like Shuanghuanglian injection (22). Mechanistically, polyphenols exert anticancer effects by inducing apoptosis, inhibiting proliferation and angiogenesis, and regulating oxidative stress and inflammation (20, 23).

Although polyphenols exhibit strong anticancer activity, their clinical translation is limited by inherently low bioavailability and chemical instability hinder clinical translation. This highlights the need for advanced delivery strategies to improve their stability, absorption, and therapeutic efficacy.

### 2.4 Alkaloid-based therapeutic agents

Alkaloids are nitrogen-containing compounds prevalent in traditional Chinese medicine, particularly in plants like *Coptis chinensis*, *Atropa belladonna*, and *Taxus* species (24). They display extensive structural and functional diversity. Bioactive compounds such as vincristine, matrine, berberine, colchicine, and paclitaxel are widely used in adjuvant cancer therapy, underscoring their therapeutic relevance (25). Paclitaxel was approved in 1993 for treating breast, ovarian, pancreatic, and prostate cancers (26). Their antitumor mechanisms include regulating the cell cycle, inducing apoptosis, and modulating immune responses to suppress tumor growth and metastasis (27).

### 2.5 Essential oil-based therapeutic agents

Essential oils are volatile, aromatic liquids rich in monoterpenes, sesquiterpenes, and their oxidized derivatives, commonly found in *Zingiberaceae* (e.g., ginger, turmeric), *Lamiaceae* (e.g., mint, rosemary), *Rutaceae* (e.g., citrus), and *Apiaceae* (e.g., celery) plants (28). Major bioactive compounds include *β*-elemene, menthone, and limonene (29). Their lipophilic nature facilitates preferential accumulation in the lipid-rich tumor microenvironment (TME), enhancing tumor targeting and anticancer activity (30). The anticancer effects of essential oils involve inducing apoptosis, inhibiting proliferation and angiogenesis, promoting DNA repair, scavenging free radicals, increasing membrane permeability, and causing membrane depolarization (31). For example, lemongrass (*Cymbopogon citratus*) oil induces apoptosis and cell cycle arrest in A549 lung cancer cells, while *Origanum majorana* oil inhibits NSCLC cell proliferation, migration, and invasion by activating caspase-3/7 and downregulating survivin (32, 33).

Some essential oils have demonstrated potential as adjuncts in cancer care. Lavender oil, for instance, has been shown to alleviate anxiety, pain, and sleep disturbances in cancer patients (34). However, the volatility, poor solubility, and gastrointestinal instability of essential oils limit their oral and topical application. Recent advances in delivery systems—such as solid lipid carriers and cyclodextrins—have significantly improved the bioavailability, stability, and tumor-targeting efficiency of essential oils. These technologies highlight their promise in precision cancer therapy, improving efficacy while minimizing side effects (35).

## 3 Mechanistic insights into FMH-derived bioactive compounds for lung cancer therapy

**Figure 1:**
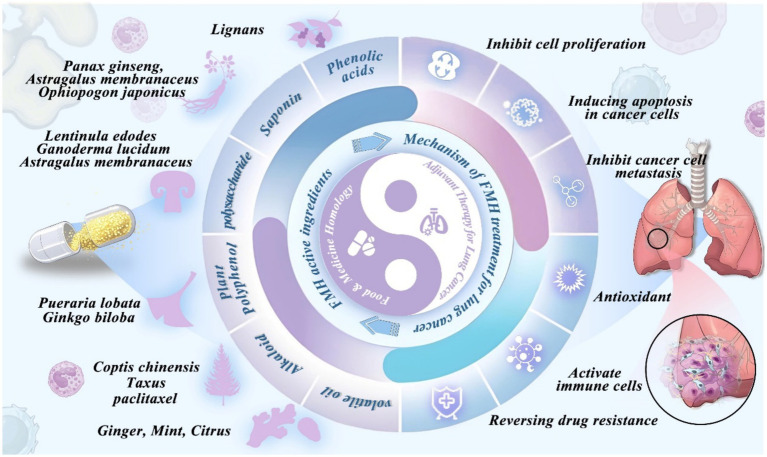
Representative bioactive compounds of food-medicine homologous (FMH) substances and their antitumor mechanisms in lung cancer therapy. Bioactive ingredients—polysaccharides, saponins, polyphenols (e.g., flavonoids, phenolic acids, lignans), alkaloids, and essential oils—extracted from medicinal and edible plants (e.g., *Panax ginseng*, *Astragalus membranaceus*, *Ganoderma lucidum*, *Pueraria lobata*, *Ginkgo biloba*, *Coptis chinensis*, *Citrus* species), exert multifaceted antitumor effects through apoptosis induction, autophagy modulation, metastasis inhibition, oxidative stress regulation, multidrug resistance reversal, and immune microenvironment remodeling.

Food-medicine homologous (FMH) substances, with their natural origin, nutritional value, and low toxicity, exhibit multi-targeted anticancer effects in lung cancer therapy. Their bioactive compounds modulate immunity, oxidative balance, and metabolism, contributing to disease control and treatment precision. For example, *Astragalus*, *Ganoderma lucidum (Curtis) P. Karst. (Lingzhi mushroom)*, and *Lonicera japonica Thunb. (Japanese honeysuckle)* enhance immune function, while *Bupleurum chinense* and *Scutellaria baicalensis* alleviate oxidative stress and shape the tumor microenvironment. These attributes support the role of FMH as nutritional adjuncts in integrated lung cancer management (36). The following sections detail their key mechanisms of action (Figure 1).

### 3.1 Inhibition of cell proliferation and cell cycle arrest

FMH possess broad multi-target and multi-pathway properties, offering considerable potential in cancer prevention and therapy. Several representative bioactive compounds modulate key oncogenic signaling cascades. For instance, FIP-fve inhibits A549 cell proliferation and migration through p53/p21 activation and suppression of Rac1 signaling (37). Chlorogenic acid binds to annexin A2, attenuates NF-κB–mediated anti-apoptotic gene expression, thereby suppressing A549 cell proliferation (38). Similarly, alkaloids from *Aconitum carmichaelii Debeaux (Prepared lateral root of Aconitum)* inhibit NSCLC cell growth by suppressing the PI3K/Akt–mTOR axis and altering glycolytic metabolism (39). Collectively, these findings highlight the mechanistic diversity and translational potential of FMH in lung cancer.

On the other hand, FMH suppress lung cancer cell proliferation by disrupting cell cycle progression and inducing phase-specific arrest. Emodin activates PPARγ and AMPKα, while ailanthone inhibits DNA replication and downregulates RPA1, both leading to G2/M arrest in A549 cells (40, 41). By modulating key cell cycle regulators and signaling pathways, FMH exert antiproliferative effects at multiple stages, supporting the development of cell cycle-targeted therapies.

### 3.2 Induction of cancer cell apoptosis and autophagy

Apoptosis is a genetically controlled process essential for eliminating damaged or malignant cells and is a major target in cancer therapy (42). FMH induce apoptosis by activating pro-apoptotic pathways and suppressing anti-apoptotic signals. Quercetin and AS-IV modulate Bcl-2/Bax expression; saffron extract activates caspase-3, −8, and −9; and luteolin induces ROS accumulation and JNK activation (43–45). Cordycepic acid further enhances apoptotic signaling by modulating the Nrf2/HO-1/NF-κB axis, while celastrol upregulates miR-24 and miR-181b and suppresses STAT3 weakening prosurvival pathways (46, 47).

In addition, FMH can induce autophagic cell death through mitochondrial dysfunction. Disruption of mitochondrial membrane potential increases the AMP/ATP ratio, activates AMPK and triggering autophagy. Ellagic acid decreases ATP levels, reduces mitochondrial potential and oxygen consumption, thereby inhibiting lung cancer cell growth (48). Polygonatum odoratum lectin promotes autophagy through Akt–mTOR inhibition, while glycyrrhetinic acid activates LC3 and the IRE1α–JNK/c-Jun axis in A549 and NCI-H1299 cells (49, 50). Luteoloside promotes autophagy through the ROS–PI3K/Akt/mTOR/p70S6K pathway, contributing to antiproliferative effects in lung cancer cells (51). Collectively, these findings illustrate the coordinated induction of apoptosis and autophagy by FMH, providing a synergistic basis for their antitumor efficacy.

### 3.3 Inhibition of tumor cell invasion and metastasis

Metastasis is the primary cause of mortality in malignant tumors and involves key steps such as invasion, extravasation, and colonization of distant organs. Natural bioactive compounds can in preventing lung cancer metastasis by inhibiting epithelial–mesenchymal transition (EMT) and modulating the extracellular matrix (ECM). Baicalin suppresses PI3K/Akt/NF-κB signaling, silymarin inhibits STAT3, ginsenoside Rh2 inactivates Wnt/*β*-catenin, and osthole targets NF-κB–Snail signaling, collectively blocking EMT and reducing migration and invasion (52–55). In addition, FMH components impede ECM degradation by downregulating matrix metalloproteinases. ZQD and AS-IV reduce MMP2 and MMP9 expression via the PI3K/Akt/p53 and PKC-*α*–ERK1/2–NF-κB pathways, respectively, thereby inhibiting ECM breakdown and lung cancer cell invasion (56, 57).

### 3.4 Antioxidant activity

Under physiological conditions, reactive oxygen species (ROS) production and clearance are tightly regulated. Excessive ROS accumulation can induce tumor cell death, making ROS modulation a promising anticancer strategy. Selective of ROS may preferentially kill cancer cells while sparing normal tissue (58). Curcumin and its analog EF24 increase ROS production, promote mitochondrial fission, and inhibit NSCLC cell proliferation, inducing apoptosis (59). Resveratrol enhances oxidative stress in lung cancer cells by downregulating Nrf2 and its downstream antioxidants (CAT, HO-1, NQO1, SOD1), thereby disrupting redox homeostasis and triggering senescence and apoptosis (60). Together, these findings highlight ROS-directed vulnerability as an key mechanism through which FMH-derived compounds exert antitumor activity.

### 3.5 Immunomodulation

Chronic inflammation promotes tumor progression by inducing immunosuppression, remodeling the tumor microenvironment (TME), and directly affecting cancer cells. Several FMH components improve the TME and enhance immunotherapy by boosting antitumor immunity and suppressing immunosuppressive cells. PG2 promotes M1 macrophage polarization, dendritic cell maturation, and T cell activation in lung cancer patients, enhancing immune responses and improving immunotherapy outcomes (61). The L-fucose moiety of GLP stimulates IgM antibody production, increasing tumor-targeted immunotoxicity. Salidroside (Sal) inhibits regulatory T cell (Treg) activation, downregulates FOXP3, and promotes CD8^+^ and effector CD4^+^ T cell infiltration, effectively countering immune evasion (62). Collectively, FMH contribute to antitumor immunity by reprogramming the TME and enhancing immune-based therapies.

### 3.6 Reversing multidrug resistance (MDR)

MDR is a major obstacle in chemotherapy, allowing tumor cells to evade agents and reducing treatment efficacy. FMH-derived bioactive compounds can reverse MDR by modulating drug efflux, aberrant signaling, and chemosensitivity.

One major cause of MDR is the overexpression of drug efflux transporters. Naringin increases cisplatin sensitivity in A549/DDP cells by upregulating Bax and downregulating P-glycoprotein (P-gp), MRP1, p-Akt, and CXCR4, reducing drug efflux (63). Ginsenoside Rb1 targets ATP-binding cassette sub-family B member 1(ABCB1) and PTCH1 via the Hedgehog pathway, suppressing efflux activity and reversing resistance (64). MDR is also driven by mutations in key signaling pathways (e.g., EGFR, AKT) and activation of compensatory mechanisms (e.g., Nrf2, HIF-1α), which promote immune evasion and apoptosis resistance. Curcumin enhances cisplatin sensitivity by inhibiting CA916798 and the PI3K/AKT pathway (65). Tangeretin reverses paclitaxel and osimertinib resistance via Nrf2/P-gp inhibition (66). Triptolide sensitizes A549 xenografts to cisplatin by downregulating Nrf2 (67). Moreover, epithelial–mesenchymal transition (EMT) confers invasive properties and resistance. Emodin reverses doxorubicin resistance in H69AR cells by downregulating EMT-related transcription factors (Twist, Snail, Slug) and NF-κB signaling (68). In summary, FMH counteract MDR by targeting efflux transporters, aberrant signaling, and EMT, offering novel adjuncts to improve chemotherapy efficacy. In summary, FMH counteract MDR by targeting efflux transporters, dysregulated signaling, and EMT, offering promising adjuncts to enhance chemotherapy efficacy.

## 4 Nanodelivery systems (NDDS) potentiate the anticancer effects of FMH in lung cancer therapy

**Figure 2:**
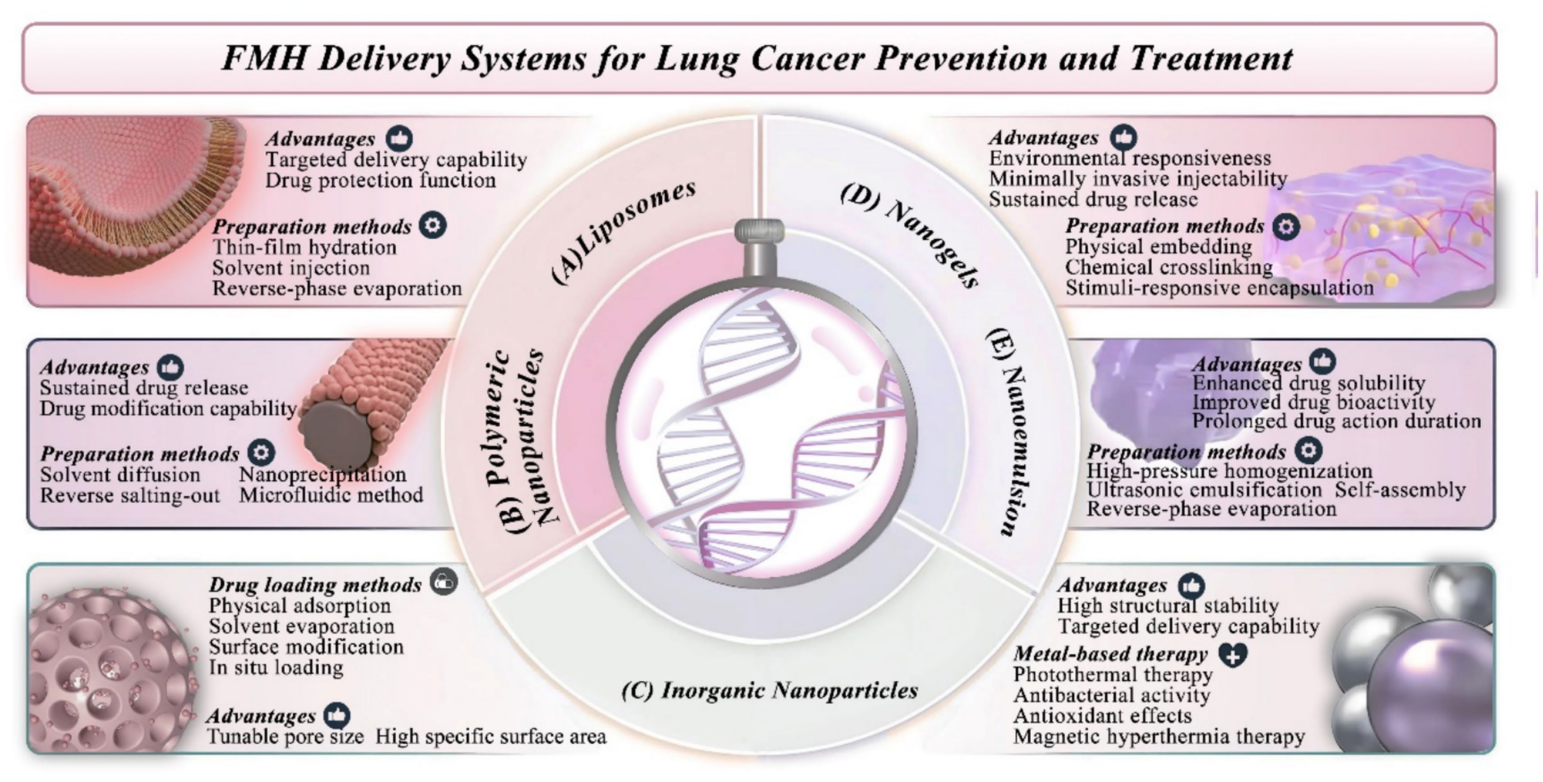
Nanodelivery strategies for enhancing the therapeutic efficacy of FMH-derived compounds in lung cancer treatment. Engineered nanocarrier platforms—liposomes (thin-film hydration, solvent injection), polymeric nanoparticles (nanoprecipitation, microfluidics), inorganic nanoparticles (mesoporous silica, gold, iron oxide), nanogels (crosslinking, ionic gelation), and nanoemulsions (high-pressure homogenization, ultrasonication)—overcome solubility, stability, and bioavailability limitations of FMH-derived bioactive compounds. These nanodelivery systems enhance targeted delivery, sustained release, and bioactivity, offering improved precision and safety in lung cancer therapy.

Building on these mechanisms, recent advances in nanodelivery systems aim to overcome the bioavailability limitations of FMH-derived compounds and enhance their therapeutic precision in lung cancer. FMH (food-medicine homologous) substances possess both therapeutic and nutritional attributes, demonstrating notable antitumor efficacy. Traditional FMH formulations include oral decoctions, powders, injections, and inhalation therapies, with the latter offering enhanced pulmonary targeting. However, their clinical application remains limited by poor stability and low bioavailability. NDDS overcome these challenges by improving absorption, distribution, and metabolic stability, thereby enhancing therapeutic efficacy. The following section summarizes the design strategies, functional characteristics, and therapeutic potential of NDDS in lung cancer (Figure 2).

### 4.1 Liposomes

Liposomes are nanosized vesicles composed of phospholipid bilayers, typically 25–2,500 nm in diameter, capable of encapsulating hydrophilic, hydrophobic, and amphiphilic drugs (69). Due to their biocompatibility, liposomes offer controlled release, targeted delivery, and protection of payloads, thereby improving pharmacokinetics (70). In pulmonary drug delivery, inhaled liposomes directly target alveoli and airway epithelium, bypassing first-pass metabolism and enhancing local concentrations. This strategy has been widely applied to treat chronic obstructive pulmonary disease (COPD), lung cancer, and pulmonary infections. Liposomal formulations significantly enhance bioavailability and efficacy, offering a promising approach to pulmonary disease management.

Liposomes are commonly prepared using thin-film hydration, solvent injection, or reverse-phase evaporation (Figure 2A). Thin-film hydration suits lipophilic drugs as shown by PTF-loaded solid lipid nanoparticles with enhanced antitumor activity and reduced toxicity (71). Reverse-phase evaporation, ideal for hydrophilic drugs, forms water-in-oil emulsions that yield liposomes upon solvent removal. Zhao et al. f produced tetrandrine lipid nanocapsules with increased oral bioavailability (72). Curcumin liposomal dry powder inhalers (LCDs), made via freeze-drying, offer rapid pulmonary action with reduced systemic toxicity (73). Drug loading—via passive or active (remote) strategies—is critical in liposome formulation. In passive loading, hydrophilic drugs are trapped in the aqueous core, while hydrophobic ones embed in the bilayer during liposome formation. However, this method has low encapsulation efficiency (≤30%), high leakage risk, and limited membrane stability. These drawbacks have led to more efficient active loading techniques (74). Active loading uses a pH or ion gradient to drive weak acids or bases (e.g., berberine, EGCG) into liposomes in their uncharged form, where they ionize and become trapped—enhancing loading and retention (75).

### 4.2 Polymeric nanoparticles (NPs)

NPs are composed of a lipid–PEG outer shell and a polymeric core, typically 1–1,000 nm in diameter, and are categorized into nanospheres, nanocapsules, and polymeric micelles (76). Nanocapsules encapsulate the drug within a polymeric shell, whereas nanospheres entrap the drug throughout a cross-linked polymer matrix. Both architectures can be functionalized via surface adsorption of bioactive compounds, broadening drug delivery (77). NPs provide tunable physicochemical properties—including size, morphology, surface charge, and degradation rate—making them amenable to scalable production (78). In the context of lung cancer therapy, NPs effectively penetrate the mucus barrier and can be readily engineered for targeted delivery to alveolar or tumor tissues. Their sustained-release profiles enhance local drug retention while minimizing systemic toxicity (79).

Nanocarriers are typically synthesized using solvent diffusion, reverse salting-out, nanoprecipitation, or microfluidic techniques (Figure 2B). Nanoprecipitation to enables curcumin-loaded micelles, with prolonged release profiles (80). Inhalable silibinin nanoparticles, formulated with poly(*ε*-caprolactone)/Pluronic F68, traverse the lung mucus barrier and preferentially accumulate in tumor tissues and sustain drug release, thereby markedly improving therapeutic efficacy against both SCLC and NSCLC. This formulation represents a promising strategy for localized lung cancer therapy (81). However, NP aggregation compromises colloidal stability, accelerates clearance by the reticuloendothelial system (RES), and may induce pulmonary inflammation. Furthermore, the tumor microenvironment (TME)—marked by hypoxia, acidic pH, and dense mucus—poses formidable barriers to NP penetration. These factors collectively challenge the efficacy of NP-mediated lung cancer therapy, highlighting the need for innovative delivery strategies (82).

### 4.3 Inorganic nanoparticles (INPs)

Common INPs—such as silica, iron oxide, gold, silver, and carbon nanotubes—exhibit structural stability, high drug-loading efficiency, and diverse physicochemical attributes, including magnetic, optical, and electrical functionalities (Figure 2C). Compared with organic nanocarriers, INPs offer enhanced tissue penetration, magnetic hyperthermia conversion capacity, and responsiveness to the acidic tumor microenvironment. These features make them particularly suitable for inhalation-based delivery, localized treatment, and real-time imaging, thereby improving the precision and effectiveness of lung cancer therapy (83).

Mesoporous silica nanoparticles (MSNPs) provide high loading efficiency; for example, curcumin-loaded SBA-15 effectively inhibit lung cancer progression (84). Carbon nanotubes, induce oxidative stress and apoptosis in A549 cells (85). Navin et al. (86) constructed magnetic core–shell nanoparticles for inhalable quercetin delivery, showing strong cytotoxicity. Metallic nanoparticles such as gold nanoparticles (AuNPs) in photothermal therapy (PTT) due to efficient photothermal conversion. Silibinin-conjugated, pH-sensitive AuNPs show enhanced cytotoxicity (87). Additionally, AuNPs function as diagnostic sensors via analysis of exhaled breath components (88). Silver nanoparticles (AgNPs) exert antibacterial, antioxidant, and anti-inflammatory activities by generating ROS and releasing silver ions. Rhododendron-derived AgNPs show potent pro-apoptotic activity (89). Nonetheless, their dose-dependent toxicity and long-term safety profiles necessitate further evaluation (90). Iron oxide nanoparticles enable magnetic hyperthermia therapy (91). *In vitro*, magnetic liposomes incorporating ferrite-based nanoflowers show robust uptake and activity against A549 cells (92). The Fe₃O₄@PLGA magnetic core–shell system, supports inhalable quercetin delivery with limited toxicity (86).

Despite their potential, INPs present challenges such as poor biodegradability, immunogenicity, and oxidative stress-associated cytotoxicity (93). Rational tuning of pore architecture and diameter may improve drug-loading efficiency, enable sustained release, and mitigate burst-related toxicity.

### 4.4 Nanogels hydrogels

Nanogels are three-dimensional networks of cross linked hydrophilic polymers that swell in aqueous environments and respond to external stimuli such as temperature, pH, and light (Figure 2D). Their structural flexibility and injectability render them ideal for pulmonary administration, enabling localized controlled release, extended retention time, and reduced systemic toxicity in lung cancer therapy. Compared with conventional hydrogels, nanogels can encapsulate nanoparticles to further augment therapeutic efficacy (94). Their stable drug encapsulation enables sustained release but their short gastrointestinal residence time limits oral delivery efficiency (95).

FMH-derived polysaccharides, flavonoids, and alkaloids are widely employed in hydrogel fabrication because of their biocompatibility and slow-release features (95). Typical fabrication strategies include physical entrapment, chemical crosslinking, and stimuli-responsive encapsulation, among which ionic gelation is preferred for its simplicity. Abbasalizadeh et al. (96) utilized CaCl₂-mediated ionic gelation to develop a curcumin-piceatannol alginate-chitosan hydrogel, which suppressed T47D and A549 proliferation and induced apoptosis. In immunotherapy, nanogels encapsulating tumor antigens and immunomodulators can counteract immune evasion and enhance antitumor immune responses. For example, a G-Rh2-loaded ICG-HPC-AA/BSA hydrogel stimulates antitumor immunity (97). Orally administered Pur nanoparticles, prepared using chitosan–tripolyphosphate crosslinking, exhibit sustained release and improved bioavailability (98). However, the porous structure of nanogels may restrict loading of macromolecules or high-dose agents, limiting their suitability for rapid-onset therapies and necessitating combination with other delivery platforms.

### 4.5 Nanoemulsion

Nanoemulsions are transparent, thermodynamically stable oil–water systems with particle sizes of 10–100 nm, formed using water, oil, surfactants, and co-surfactants (99). They improve drug solubility and bioavailability, while exhibiting low toxicity and high biocompatibility (100). Fabrication methods include high-energy techniques (e.g., high-pressure homogenization, ultrasonication) and low-energy approaches (e.g., self-assembly, reverse-phase evaporation). High-energy methods are suitable for heat-stable drugs, whereas low-energy approaches are preferred for thermosensitive compounds(Figure 2E). Curcumin microemulsions enhance solubility and bioactivity, exhibiting strong cytotoxic effects against A549 and HepG2 cells; when combined with cisplatin, they improve liver function and reduce oxidative stress (101). A cationic nanoemulsion of Brucea javanica oil (BJO) improved its oral bioavailability and exhibited synergistic antitumor activity with vinorelbine (102). Nanoemulsions also prolong circulation and support targeted delivery. However, limited hemocompatibility—especially in positively charged formulations—remains a concern due to their tendency to induce platelet aggregation. To reduce thrombosis risk, surface PEGylation or integration of neutral/negatively charged components is employed to enhance biocompatibility and safety (103). Modifying excipients like phospholipids and cholesterol further minimizes coagulation risk.

## 5 Targeting the gut–lung axis: FMH-based strategies in lung cancer therapy

**Figure 3:**
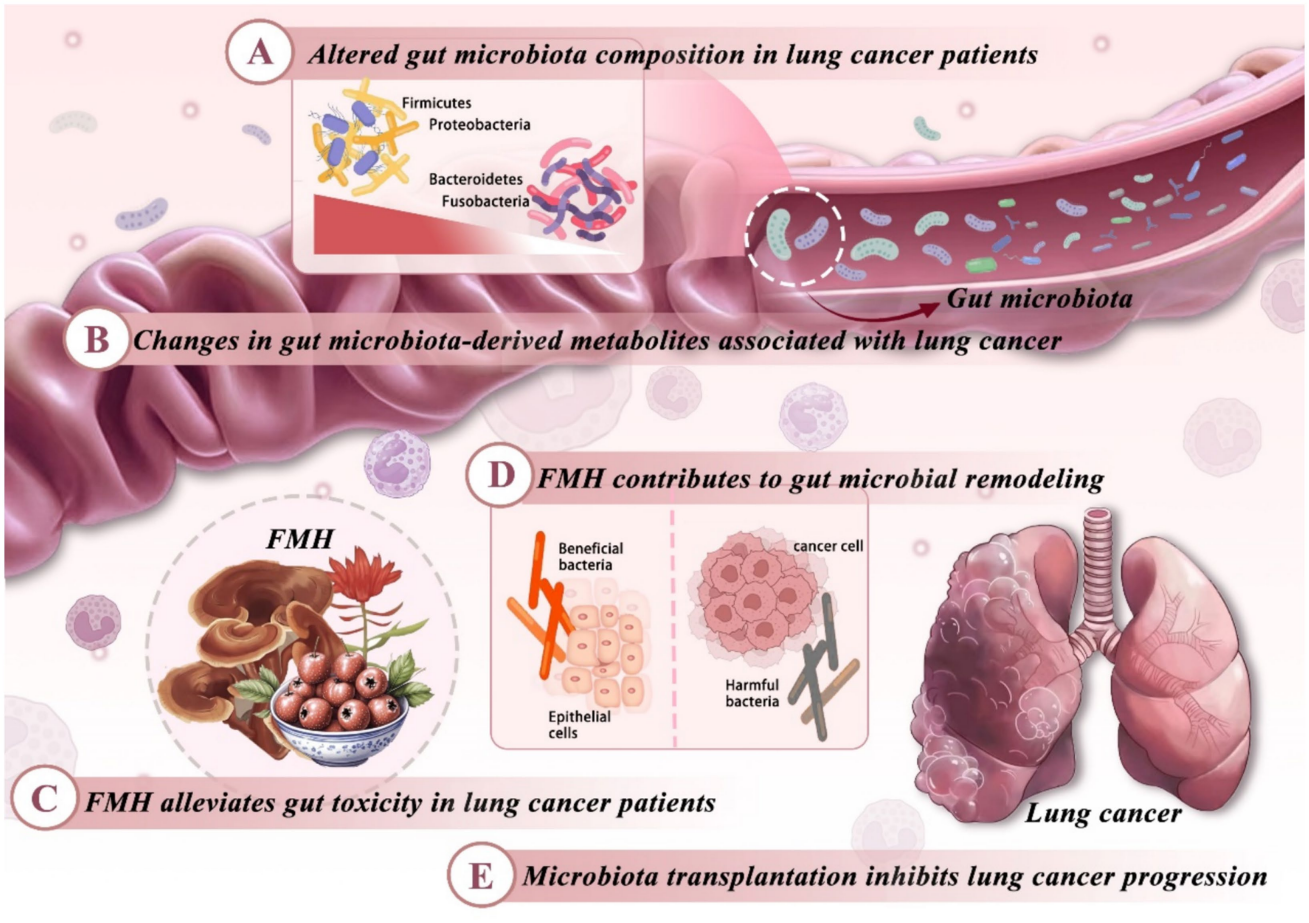
Regulatory mechanisms of FMH substances on the gut–lung axis in lung cancer therapy. Lung cancer alters gut microbiota composition, notably decreasing Firmicutes and increasing Bacteroidetes, Proteobacteria, and Fusobacteria, which disrupts metabolite production (e.g., SCFAs, bile acids) that influence tumorigenesis and immunity. FMH substances restore microbial equilibrium, reinforce epithelial barriers, downregulate pro-inflammatory cytokines, enrich beneficial taxa (e.g., *Akkermansia*, *Lactobacillus*), and suppress pathogens, thereby enhancing systemic immunity and modulating the tumor microenvironment. Interventions such as FMT and FMH-driven microbiota remodeling further highlight the gut–lung axis as a therapeutic target in lung cancer.

Beyond physicochemical delivery enhancements, targeting host–microbiota interactions through the gut–lung axis provides an additional avenue to potentiate FMH-based interventions. In recent years, the “gut–lung axis” has emerged as a critical bidirectional communication pathway linking the gut microbiota with respiratory immune regulation, and has become increasingly relevant to lung cancer research. Mounting evidence indicates that the lung and gut originate from the same embryonic foregut and engage in dynamic crosstalk mediated by microbiota, microbial metabolites, and immune signaling pathways. This complex interplay contributes to lung cancer initiation and progression, underscoring the important role of microbiota-mediated mechanisms in tumor pathogenesis (104). Dysbiosis of the gut microbiota perturbs intestinal immune homeostasis and alters systemic immunity via microbial metabolites such as short-chain fatty acids (SCFAs), thereby shaping the pulmonary immune microenvironment (Figure 3).

### 5.1 Alterations in gut microbiota profiles in patients with lung cancer

The gut microbiota of lung cancer patients exhibits notable dysbiosis, marked by a reduced abundance of Firmicutes and Proteobacteria, and an enrichment of Bacteroidetes and Fusobacteria (Figure 3A). Firmicutes exert anti-inflammatory effects and modulate T cell activity via the NF-κB signaling pathway; their depletion may compromise mucosal immune defense (105). Specific genera within Bacteroidetes, particularly *Bacteroides*, are linked to CTLA-4 and PD-1 signaling pathways, and their increased prevalence may enhance immunotherapeutic responses (106). Conversely, Proteobacterial overgrowth signifies microbial imbalance and may facilitate infection and immune dysregulation (107). Pathogenic *Fusobacterium* species can upregulate proinflammatory cytokines (IL-1β, IL-6) and matrix metalloproteinases (MMPs), thereby fostering chronic inflammation and driving tumor progression.

### 5.2 Experimental studies on gut microbiota-based therapies for lung cancer

Preclinical studies suggest that gut microbiota modulation offers a novel strategy for attenuating lung cancer progression. *Akkermansia muciniphila* can colonize lung tumors, reinforce intestinal barrier integrity, and reshape gut microbial composition. These effects contribute to its antitumor potential, supporting microbiota modulation as an adjunct to lung cancer therapy (108). Fecal microbiota transplantation (FMT) also indicates a causal link between gut dysbiosis and lung cancer progression (109). FMT from NSCLC patients exacerbates pulmonary inflammation and suppresses T cell function in animal models (110). *Bifidobacterium* ferments dietary fibers to produce SCFAs, maintains immune balance, and represents a promising microbial target for intervention. Supplementation with *Bifidobacterium* or transplantation of healthy microbiota significantly inhibits bilateral lung metastasis (Figure 3B). Clinically, *Clostridium butyricum* supplementation has been associated with improved progression-free survival (111). Gut microbiota or metabolite modulation holds promise for augmenting immunotherapy efficacy in lung cancer, though mechanisms remain incompletely understood. Microbiota profiles are shaped by experimental design, cancer stage, sequencing platform, and sample size, with small cohorts prone to bias. Host-related factors—such as ethnicity, lifestyle, geography, antibiotic exposure, and immune status—also significantly influence microbiota composition. Future efforts should emphasize large-scale, multicenter studies with standardized analytical protocols to clarify the microbiota’s role in lung cancer pathogenesis.

### 5.3 Food-medicine homologous (FMH) compounds regulate gut microbiota and metabolites

Multiple FMH-derived components improve the immune microenvironment in lung cancer by reshaping gut microbiota composition and metabolic activity. Polysaccharides from *Aconitum carmichaelii Debeaux (Aconiti Lateralis Radix Praeparata)* and *Ganoderma lucidum (Curtis) P. Karst. (Lingzhi mushroom)* promote the proliferation of beneficial bacteria (*Firmicutes*, *Lactobacillus*) and suppress harmful taxa (*Proteobacteria*, *Ruminococcus*). Such modulation alleviates intestinal inflammation and restores microbial balance, contributing to improved antitumor immune responses in lung cancer (Figure 3C) (112, 113). *Salvia miltiorrhiza Bunge (Danshen)* and *Lonicera japonica Thunb. (Japanese honeysuckle)* reduce immune evasion by downregulating IL-6, TNF-*α*, and IL-1β (114, 115). Combination therapies, such as Xihuang Pill plus anlotinib, enrich *Bacteroidaceae* and *Pasteurellaceae* and inhibit tumor progression in murine lung cancer models (116). Furthermore, bioactive compounds such as baicalein, curcumin, and quercetin modulate SCFA, bile acid, and indole derivative production, to enhance the immune landscape and immunotherapy efficacy (Figure 3D). Notably, baicalin enriches *Akkermansia* and *Clostridia*, elevates SCFA levels, enhances the CD8^+^ T cell/Treg ratio, and mitigates PD-1 resistance, thereby improving antitumor immunity and therapeutic efficacy in lung cancer (117). Ginseng polysaccharides (GPs), when combined with αPD-1 therapy, elevate branched-chain fatty acids (e.g., valeric acid), maintain T cell metabolic homeostasis, and modulate the kynurenine/tryptophan (Kyn/Trp) pathway (118). *Ganoderma lucidum (Curtis) P. Karst. (Lingzhi mushroom)* polysaccharides enhance butyrate synthesis, activate the Nrf2/HO-1 axis to reduce ROS accumulation, and regulate macrophage polarization toward the M1 phenotype (119). Bufei Xiaojie Yin (BFXJY) modulates inflammation and the Kyn/Trp ratio via the PKC–NLRP3 signaling pathway, supporting microbial–immune equilibrium (120). Microbial metabolites thus represent central mediators of host–microbiota immune crosstalk and key targets for FMH-based immunomodulation. Recent studies highlight that FMH-derived polysaccharides and polyphenols modulate the gut–lung axis by targeting specific microbial taxa and immune pathways. *Faecalibacterium prausnitzii*, *Akkermansia muciniphila*, and *Lactobacillus* species, enriched following FMH supplementation, promote intestinal homeostasis and reduce systemic inflammation. These microbial shifts are associated with increased production of short-chain fatty acids such as butyrate and propionate, which help regulate the balance between regulatory T cells and Th17 cells, thereby shaping pulmonary immune responses. Meanwhile, FMH bioactive compounds including berberine and curcumin inhibit the TLR4 and NF-κB signaling pathways in gut and lung epithelial cells, resulting in decreased pro-inflammatory cytokine release. In addition, polysaccharide-rich FMH extracts from sources such as *Ganoderma lucidum (Curtis) P. Karst. (Lingzhi mushroom)* and *Astragalus membranaceus (Fisch.) Bunge (Huangqi)* promote macrophage polarization toward the anti-inflammatory M2 phenotype, contributing to an immune microenvironment less conducive to tumor progression.

### 5.4 FMH in mitigating lung cancer treatment toxicities via gut microbiota

Conventional therapies—such as chemotherapy, targeted agents, and antibiotics—often disrupt gut microbiota homeostasis, leading to immunosuppression and systemic toxicities. This dysbiosis compromises immune competence and exacerbates treatment-related toxicities, underscoring the need for microbiota-preserving or -restoring strategies to improve therapeutic efficacy and minimize toxicity (Figure 3E). In contrast, FMH alleviates toxicity through coordinated modulation of the gut microbiota, immune responses, and epithelial barrier integrity. For instance, Shenglin Decoction (SLD) increases *Akkermansia muciniphila* abundance and regulates SCFA metabolism, thereby attenuating radiotherapy-induced lymphopenia. Sijunzi Decoction (SJZD) restores postoperative SCFA metabolic balance and facilitates recovery (121). In immune modulation, glycyrrhiza flavonoids (GLA) promote anti-inflammatory M2 macrophage polarization, thereby alleviating doxorubicin-induced cardiotoxicity (122). Sijunzi San (SSG) modulates TAM polarization and downregulates MMP9 and LOX expression, thereby inhibiting lung metastasis (123). For barrier protection, ginsenosides attenuate cyclophosphamide-induced intestinal permeability and epithelial injury (124). Bufei Huayu Formula (BFHY) mitigates cisplatin-induced enteritis and reduces levels of pro-inflammatory cytokines (125). Collectively, FMH enhances treatment tolerance and patient quality of life in lung cancer by orchestrating microbiota modulation, inflammation control, and toxicity reduction.

## 6 Clinical and translational advances of FMH in lung cancer therapy

Driven by ongoing research, FMH substances have progressed from preclinical validation to clinical application in lung cancer, offering promise in overcoming drug resistance, improving chemotherapy tolerance, enhancing immune responses, and managing cancer-related fatigue (CRF) and cachexia through nutrition-linked mechanisms. A Phase I trial (NCT02321293) confirmed the safety of CURCUViva™ (curcumin) with EGFR-TKI therapy, while a Phase III study (NCT02929693) showed that the Chinese herbal formula FZYA enhanced targeted therapy by modulating the PI3K/Akt pathway (126). Astragalus-based formulations combined with chemotherapy improved survival and tumor control (ChiCTR1800019396) (127). FMH has also gained attention as an adjuvant to lung cancer immunotherapy. FMH-derived polysaccharides, such as those from Ginseng and *Astragalus sinicus*, have shown nutritional immunomodulatory effects, enhancing CD4^+^/CD8^+^ T cell ratios and promoting postoperative immune recovery (128, 129). Mechanistically, several FMH-derived bioactive compounds, including ginsenosides and curcumin, may reverse drug resistance by regulating ATP-binding cassette transporters, particularly through reducing the expression and function of P-glycoprotein. In addition, these compounds have been reported to suppress cancer stemness-associated signaling pathways such as Wnt/*β*-catenin, and to restore apoptotic sensitivity by activating the mitochondrial apoptosis cascade. These multifaceted mechanisms support the role of FMH substances not only as nutritional supplements but also as functional agents that contribute to overcoming therapeutic resistance in lung cancer.

Lung cancer–related CRF and cachexia are often associated with impaired metabolic homeostasis and malnutrition. FMH-based interventions, rooted in nutritional support and metabolic regulation, have shown benefits in alleviating these conditions (130). For instance, JPSS cream and Ganoderma–Ligustrum formulas improve energy, immunity, and daily function (131, 132). Yifei Sanjie Pill (YFSJ) relieves cisplatin-induced fatigue by modulating the AMPK/mTOR axis (133). In cachexia, where current nutritional therapies show limited efficacy, FMH such as Rikkunshito and Paeoniflorin have been found to enhance appetite, reduce inflammation, preserve muscle mass, and restore nutritional status (134, 135). Shashen Maidong Decoction increases albumin and lowers inflammatory markers, contributing to better clinical outcomes (136). Overall, FMH alleviate CRF and cachexia by regulating immunity, inflammation, and metabolism, improving quality of life in lung cancer patients.

EGFR-TKI-induced diarrhea can lead to dehydration and electrolyte imbalance, compromising both treatment efficacy and nutritional status, and reducing patient adherence. Conventional approaches—such as nutritional support, antidiarrheal agents, and dose modifications—have shown limited benefit in some cases and may cause additional side effects, complicating toxicity management (137). FMH-based interventions offer a nutrition-integrated strategy to restore gastrointestinal function and systemic balance. For example, a traditional formula containing Ginseng, Atractylodes, and Poria alleviates diarrhea by improving spleen deficiency, enhancing digestive function, and supporting nutrient absorption and fluid balance, with favorable safety outcomes (138).

Despite encouraging findings, FMH application is limited by small samples, short follow-ups, and the lack of large-scale RCTs. Future studies should apply nutritional systems pharmacology to clarify mechanisms, refine dosing, and explore synergy with immuno- and targeted therapies, advancing FMH as evidence-based nutrition-integrated strategies in lung cancer care. Current progress can be tracked via the clinicaltrials.gov database (Table 1).

**Table 1:** Clinical trials of foods and medicinal substances.

Study ID/Phase	Patient group/enrollment	Major constituents	Primary outcome measure	Study purpose
NCT02929693(III)	LUAD (*n* = 198)	Huangqi, Pseudostellaria heterophylla, Polygonatum sibiricum, Atractylodes macrocephala, Poria cocos, Rehmannia glutinosa, *Ophiopogon japonicus*, *Asparagus cochinchinensis*, *Eclipta prostrata*, *Ligustrum lucidum*, *Hedyotis diffusa*, Scutellaria barbata, *Taraxacum mongolicum*, *Cirsium setosum*	PFS	Evaluate combination efficacy
NCT06674252(II)	Patients with symptoms like cough, shortness of breath, and fatigue after lung cancer resection (n = 174)	Huangqi, *Codonopsis pilosula*, Schisandra chinensis, Rehmannia glutinosa, Ziyuan, Mulberry bark, Snake berry, Half branch lotus, White *Hedyotis diffusa*, Bai Qian, *Platycodon grandiflorum*	MDASI-TCM scale	Assess symptom relief post-surgery
JAPIC CTI-142747(II)	Lung cancer (*n* = 60)	Atractylodes lancea rhizome, Ginseng, Pinellia tuber, Poria sclerotium, Jujube, Citrus unshiu peel, Glycyrrhiza, Ginger	Change in dietary intake from day 0 to day 7 of chemotherapy	Evaluate anorexia relief from TJ-43
ChiCTR-IOR-14005679(−)	NSCLC patients carrying EGFR exon 19 deletions or L858R mutations (*n* = 70)	Huangqi, *Ligustrum lucidum*, Ganoderma lucidum, Dioscorea opposite, *Hedyotis diffusa*, *Prunella vulgaris*	PFS	Assess gefitinib enhancement and safety.
NCT02321293(I)	EGFR-mutant NSCLC (unresectable stage 3A, 3B, or stage 4) (*n* = 20)	Curcumin	Feasibility (patient willingness and adverse events)	Assess safety of curcumin + EGFR-TKIs
NCT06387134(−)	NSCLC (*n* = 40)	Ginseng, Blackhead, Rhubarb, Aster, Forehu, Thin on	Lung Cancer Quality of Life (FACT-L)	Evaluate Lifei Xiaoji Wan efficacy
NCT02844114(II)	NSCLC (*n* = 60)	Ganoderma Lucidum Spore	FACT-G (life quality score)	Evaluate Ganoderma + chemo outcomes
NCT02195232(II/III)	NSCLC, colorectal, pancreatic adenocarcinoma (*n* = 64)	Isoquercetin	Cumulative incidence of VTE	Assess VTE prevention by Isoquercetin
NCT01317953(I)	ED-SCLC (*n* = −)	EGCG	-	Evaluate EGCG safety post-first-line
NCT02577393(II)	NSCLC (*n* = 83)	EGCG	Reduce Grade II esophagitis (RTOG score)	Assess EGCG in radiotherapy
NCT03486496(II)	NSCLC patients carrying EGFR exon 19 deletions or L858R mutations (*n* = 50)	Berberine	PFS	Assess gefitinib + Berberine efficacy
NCT01802021(II/III)	NSCLC (*n* = 120)	Astragalus	OS	Assess immunomodulation by Qingshu-Yiqi-Tang
NCT01048983(I/II)	NSCLC (*n* = −)	Curcumin	Combined AUC for symptoms	Supportive care study
ChiCTR1900023451(III)	Stage III/IV NSCLC (*n* = 60)	*Codonopsis pilosula*, Carapax Trionycis	Piper fatigue scale score (RPFS)	Evaluate JPSS paste for fatigue
JPRN-UMIN000006892(−)	Advanced/recurrent NSCLC (*n* = 12)	Curcumin	Safety and tolerability	Assess Ceracurmin + Erlotinib safety
NCT00573885(II)	Heavy smokers and lung cancer patients (*n* = 53)	Green tea catechin extract (defined)	Oncogene/tumor suppressor gene expression	Cancer prevention study
NCT00611650(II)	Heavy smokers and pre-cancerous lung cancer patients (*n* = 23)	Green tea catechin extract (defined)	Dysplasia severity change in bronchial biopsy	Cancer prevention study
NCT01481818(I/II)	Stage III NSCLC and Stage I breast cancer (*n* = 15)	EGCG	Number of participants with adverse events	Cancer prevention study
NCT06249854(II)	NSCLC (*n* = 70)	*Astragalus membranaceus*, *Atractylodes macrocephala*, *Panax ginseng*, *Angelica gigas*, *Ziziphus jujuba* var. inermis, *Bupleurum falcatum*, *Citrus unshiu*, *Glycyrrhiza glabra*. Cimicifuga heracleifolia, *Zingiber officinale*	PFS	Evaluate efficacy

AUC, area under the curve; EGCG, epigallocatechin gallate; FACT-G, Functional Assessment of Cancer Therapy–General; FACT-L, Functional Assessment of Cancer Therapy–Lung; NSCLC, non-small cell lung cancer; OS, overall survival; PFS, progression-free survival; VTE, venous thromboembolism.

## 7 Conclusion and perspectives

The concept of “Food is Medicine” has gained broad recognition in modern medicine, with growing evidence supporting the role of dietary and nutrition-based adjuvant therapy in maintaining health and managing chronic diseases, including cancer. As highlighted by *Nature Medicine*, nutrition is not only foundational for overall health but also a key modifiable factor in disease prevention and progression. Many countries have integrated dietary guidelines into public health policies, emphasizing nutrition as a strategy for cancer prevention, recurrence reduction, and prognosis improvement. Epidemiological studies show that plant-based diets, low intake of refined carbohydrates, and adherence to the Mediterranean diet are significantly associated with a lower risk of lung cancer (139, 140). FMH substances, characterized by their dual nutritional and therapeutic functions, bridge the gap between diet-based prevention and medical treatment, offering innovative, nutrition-driven strategies for the comprehensive management of lung cancer.

In the adjunctive treatment of lung cancer, FMH exert multifaceted antitumor effects through nutrition-linked immunomodulation, tumor microenvironment remodeling, reversal of drug resistance, and enhancement of systemic antitumor immunity. Emerging evidence also underscores their role in modulating gut microbiota and activating the gut–lung axis, highlighting the importance of microbiota–nutrition–immunity crosstalk, which may contribute to improved clinical responses in selected immunotherapy settings, such as PD-1/PD-L1 blockade. Furthermore, the incorporation of nanodelivery systems has shown promising potential to enhance the bioavailability and tissue-specific targeting of FMH-derived bioactive compounds, thereby contributing to improved therapeutic precision in cancer treatment. Nevertheless, the pathway to clinical translation warrants thorough evaluation, particularly in terms of achieving lung-selective accumulation, ensuring the long-term biosafety of carrier materials such as liposomes and biodegradable polymers, and addressing technical challenges associated with scalable, GMP-compliant manufacturing processes.

Importantly, the synergistic interplay between the nutritional attributes such as vitamins, trace elements and amino acids and the pharmacological actions including anti-inflammatory, immunomodulatory and anticachectic effects of FMH-derived compounds warrants greater emphasis. Unlike conventional nutritional support such as oral nutritional supplements, which mainly provide energy and basic nutrients, FMH substances can actively modulate tumor metabolism, systemic inflammation and muscle wasting. These dual-function properties may offer added benefits in cancer cachexia management. Comparative studies with standard nutritional strategies could further demonstrate their clinical value.

Despite these advances, the clinical translation of FMH faces challenges related to complex compositions, standardization difficulties, and a lack of high-quality nutritional and clinical evidence. While substantial new international references have been added in response to reviewer suggestions, we recognize that the current synthesis still cannot fully capture the rapidly expanding spectrum of FMH-mediated anticancer mechanisms, which represents an inherent limitation of this review. Most current studies remain at the preclinical stage, and even the few available clinical trials are limited by small sample sizes, short follow-up durations, and insufficient control of confounding factors such as baseline dietary patterns, lifestyle heterogeneity, and gut microbiota variability. Future research should prioritize the systematic evaluation of dose–response relationships, treatment regimens, and delivery strategies for FMH-derived compounds through multicenter clinical trials. It is particularly important to distinguish nutritional from pharmacological thresholds. Several bioactive compounds, such as curcumin, have been shown to exert beneficial antioxidant or anti-inflammatory effects at low concentrations, but may induce pro-oxidative or cytotoxic responses at higher doses. Therefore, defining safe and effective dosage windows will be essential for clinical translation. Moreover, multi-omics approaches, particularly the integration of metabolomics and metagenomics, could help identify FMH-responsive populations and elucidate mechanistic pathways underlying variable therapeutic responses. In parallel, individualized microbiota-targeting strategies, such as customized probiotics or prebiotics tailored to the patient’s baseline microbial and metabolic profile, may enhance the precision and effectiveness of FMH-based interventions.

Collectively, FMH-based therapies hold considerable promise for improving lung cancer outcomes through multi-target regulation and nutrition-mediated microbiota modulation. Bridging traditional medicine with modern nutritional pharmacology offers new opportunities for developing safe, personalized, and effective integrative strategies in thoracic oncology.
